# Distinct multimodal biological and functional profiles of symptom-based subgroups in recent-onset psychosis

**DOI:** 10.21203/rs.3.rs-3949072/v1

**Published:** 2024-03-13

**Authors:** Nikolaos Koutsouleris, Madalina-Octavia Buciuman, Clara Sophie Vetter, Clara Francesca Charlotte Weyer, Paul Zhutovsky, Santiago Tovar Perdomo, Adyasha Khuntia, yuri milaneschi, David Popovic, Anne Ruef, Dominic Dwyer, Katharine Chisholm, Lana Kambeitz, Linda Antonucci, Stephan Ruhrmann, Joseph Kambeitz, Anita Riecher-Rössler, Rachel Upthegrove, Raimo Salokangas, Jarmo Hietala, Christos Pantelis, Rebekka Lencer, Eva Meisenzahl, Stephen Wood, Paolo Brambilla, Stefan Borgwardt, Alessandro Bertolino, Peter Falkai

**Affiliations:** Ludwig-Maximilians-University; Ludwig-Maximilians-University; Ludwig-Maximilians-University; Ludwig-Maximilians-University; Amstedam Medical Center; Ludwig-Maximilians-University; Amsterdam UMC, Vrije Universiteit/GGZ inGeest; Amsterdam UMC, Vrije Universiteit/GGZ inGeest; Ludwig-Maximilians-University; LMU; Aston University; Aston University; University of Cologne, Faculty of Medicine and University Hospital; Faculty of Medicine and University Hospital, University of Cologne, Cologne; Faculty of Medicine and University Hospital, University of Cologne, Cologne; Faculty of Medicine and University Hospital, University of Cologne, Cologne; University of Basel; University of Birmingham; University of Turku; University of Turku; University of Melbourne; University of Melbourne; University of Melbourne; University of Melbourne; University of Milan; University of Lübeck; Group of Psychiatric Neuroscience, Department of Basic Medical Science, Neuroscience and Sense Organs, Aldo Moro University, Bari; University Hospital LMU

**Keywords:** gray matter volume, polygenic risk score, neurocognition, factor analysis, functioning trajectories, early psychosis

## Abstract

Symptom heterogeneity characterizes psychotic disorders and hinders the delineation of underlying biomarkers. Here, we identify symptom-based subtypes of recent-onset psychosis (ROP) patients from the multi-center PRONIA (Personalized Prognostic Tools for Early Psychosis Management) database and explore their multimodal biological and functional signatures. We clustered N = 328 ROP patients based on their maximum factor scores in an exploratory factor analysis on the *Positive and Negative Syndrome Scale* items. We assessed inter-subgroup differences and compared to N = 464 healthy control (HC) individuals regarding gray matter volume (GMV), neurocognition, polygenic risk scores, and longitudinal functioning trajectories. Finally, we evaluated factor stability at 9- and 18-month follow-ups. A 4-factor solution optimally explained symptom heterogeneity, showing moderate longitudinal stability. The ROP-MOTCOG (*Motor/Cognition*) subgroup was characterized by GMV reductions within salience, control and default mode networks, predominantly throughout cingulate regions, relative to HC individuals, had the most impaired neurocognition and the highest genetic liability for schizophrenia. ROP-SOCWD (*Social Withdrawal*) patients showed GMV reductions within medial fronto-temporal regions of the control, default mode, and salience networks, and had the lowest social functioning across time points. ROP-POS (*Positive*) evidenced GMV decreases in salience, limbic and frontal regions of the control and default mode networks. The ROP-AFF (*Affective*) subgroup showed GMV reductions in the salience, limbic, and posterior default-mode and control networks, thalamus and cerebellum. GMV reductions in fronto-temporal regions of the salience and control networks were shared across subgroups. Our results highlight the existence of behavioral subgroups with distinct neurobiological and functional profiles in early psychosis, emphasizing the need for refined symptom-based diagnosis and prognosis frameworks.

## 1. Introduction

Psychosis is an umbrella term for diverse and dynamically evolving clinical phenotypes that affect the processing of perceived information about reality. This heterogeneity hinders the development of optimal biologically informed early diagnostic and therapeutic tools in line with the scope of precision psychiatry [1], [2]. As an alternative to the classic psychiatric nosology (e.g., Diagnostic And Statistical Manual Of Mental Disorders, [3]), approaches such as the Research Domain Criteria (RDoC, [4]) or the Hierarchical Taxonomy Of Psychopathology (HiTOP, [5]) propose focusing on lower-order, symptom-based disease constructs, which may be more closely related to specific neurobiological pathways.

In schizophrenia, the distinction between positive (e.g., hallucinations, delusions), negative (e.g., blunted affect, avolition, anhedonia), and cognitive/general symptoms (e.g., disorganized speech and thought) has proven helpful in delineating the symptom complexity observed in clinical practice [6], [7], based on widely used scales such as the *Positive and Negative Syndrome Scale* (PANSS, [8]). In the early stages of psychosis, four to five PANSS symptoms-based subgroups have been commonly reported [7], [9]–[14], with symptom dimensions shown to dynamically evolve [15], [16]) and associate with functional ([10], [14]) and treatment outcomes (Martinuzzi et al., 2019).

Importantly, psychotic symptom dimensions have been associated with different layers of biological variability, suggesting their proximity to pathophysiological processes, in line with RDoC. Specifically, neurocognitive functions closely linked to specific brain networks [17]–[19] are mainly associated with negative symptoms in early-stage psychosis [20]–[23]. Furthermore, symptom-dependent structural brain abnormalities have been reported in patients with established schizophrenia [24]–[27]. For instance, Koutsouleris et al. (2008) [24] found that patients with predominantly positive, negative, or disorganized symptoms, as identified using a PANSS-factorization approach, showed distinct patterns of gray matter density reductions but also cross-subtype alterations within prefrontal-perisylvian regions. Evidence for relationships between gray matter volume (GMV) alterations and specific symptom categories also exists in patients with first-episode psychosis [28]–[34], pointing towards multiple potential neurobiological pathways that pleiotropically map to psychosis.

Moreover, the genetic liability for schizophrenia has been differentially associated with symptom dimensions both in patients with chronic schizophrenia [35], [36] and in first-episode psychosis [37], [38]. Specifically, significant associations between polygenic risk scores for schizophrenia and both negative [38], positive [38], and general PANSS-based symptoms [37] were reported in first-episode psychosis by different studies, highlighting the complex and still insufficiently delineated associations between genetic factors and distinct symptom constellations.

Despite the existing evidence that psychotic symptom dimensions are differentially associated with biological signatures, we currently lack a comprehensive multi-layer characterization of early-stage symptom-based psychosis subgroups. Here, we aimed to address this gap by exploring multimodal biological signatures of symptom-based subtypes of patients with recent-onset psychosis (ROP) from the multi-site European PRONIA study (Personalized Prognostic Tools for Early Psychosis Management; https://www.proniapredictors.eu/pronia/index.html). Concretely, we examined (1) the PANSS-based symptom structure within ROP patients and its longitudinal stability over 9- and 18-month follow-up time points using a data-driven factorization approach, (2) characterized patient subgroups defined by their most pronounced symptom dimension in terms of neurocognition, GMV, and polygenic risk scores (PRS) to obtain a deep multimodal delineation of disease heterogeneity in early illness stages, and (3) compared longitudinal functioning trajectories of the obtained patient subgroups to assess their prognostic value.

## 2. Methods

### 2.1 Study Participants

The total sample consisted of 328 ROP patients and 464 HC individuals (see consort chart, Figure S1), recruited across nine sites in Finland, Germany, Italy, Switzerland, and the United Kingdom as part of the longitudinal, multi-site PRONIA study. For assessing the longitudinal stability of the factor analysis solution, we used data collected at three time points: baseline (T0), 9-month (T1), and 18-month follow-up (T2). For further information on the PRONIA project, including inclusion and exclusion criteria, see Table S1. Adult participants gave written informed consent, and patients younger than 18 and their guardians gave written informed assent and consent, respectively. The study was registered at the German Clinical Trials Register (DRKS00005042) and approved by the research ethics committees of all sites.

### 2.2 Exploratory factor analysis and factor model selection

We analyzed patterns of co-occurring symptoms at baseline (T0) by employing a maximum-likelihood-based exploratory factor analysis (EFA) of the PANSS [8] items using R [39], R Studio [40], and the *psych* (2.1.6) package in R [41]. EFA is a dimensionality reduction technique that aims to find latent dimensions underlying the observed data [42]. As factor loadings depend on the specified number of factors, we compared solutions with one to five factors. To account for correlated symptom dimensions, we used the oblique promax rotation method [43]. We selected the best-fitting model based on the lowest average Bayesian Information Criterion (BIC) after jackknife resampling (n = 327) [44]. The BIC is a measure of model fit that introduces a penalty term relative to the number of parameters in the model [45], addressing the issue that more complex models are more prone to overfitting [46]. Jackknife resampling was employed to evaluate the stability of the factor solutions and control for sampling biases on model fit estimates [47]. We tested significant differences between models using paired t-tests and the Mann-Whitney U test between the BIC distributions derived during jackknife resampling. To investigate the stability of the factors over time, we computed the Pearson correlation coefficients (*r*) between item factor loadings across time points and assessed the percentage of items loading highest on the same factor at the different time points using the dice coefficient.

ROP patients were assigned to subgroups via majority vote during jackknife resampling based on their maximum score on the symptom dimensions of the best-fitting model. Assignment certainty was quantified by how often a subject was assigned to a group relative to another group during jackknife resampling. We assessed the longitudinal stability of the identified symptom dimensions (factor scores) through the relative number of patients assigned to the same symptom group at T1 and T2 respectively, by applying the optimal factor model found at T0, and the Pearson correlations of factor dimensions across time points.

### 2.3 Acquisition and preprocessing of structural MRI data

Neuroimaging data was available for 328 ROP patients and 464 HC individuals. The PRONIA study aimed to capture the natural heterogeneity of MRI sequences encountered in clinical settings and, therefore, required minimal harmonization of magnetic resonance imaging (MRI) scanners across sites. The T1-weighted structural sequence involved (1) acquisition in 1mm^3^ resolution, (2) enforcement of Field of View parameters that ensured a full 3D coverage of the brain, (3) maximization of the signal-to-noise ratio by choice of the relaxation- and echo-time and other related parameters (for details refer to Table S2).

At each study site, acquired T1-weighted images were visually inspected, anonymized, and defaced using an in-house script based on the Freesurfer toolbox. Subsequently, MRI scans were pre-processed with the open-source CAT12 toolbox (version r1207; http://dbm.neuro.uni-jena.de/cat12/), an extension of SPM12 using a standardized pipeline. Images were segmented into white matter, gray matter, and cerebrospinal fluid and registered to the MNI-152 space using the DARTEL algorithm [48]. Gray matter tissue maps were modulated using the Jacobian determinants from the registration step, producing GMV maps in standard space. These GMV maps were smoothed using a 4mm^3^ full-width-at-half-maximum Gaussian kernel (for details regarding these steps, see Supplementary Methods). Image quality checks were done by applying CAT12’s quality assurance framework [49], which produces a score from excellent (A) to failed (F) for each image. Images of nine participants with a score of C (satisfactory) or lower were excluded from the analysis.

### 2.4 Subgroup characterization: Neurocognition, clinical and sociodemographic data

Neurocognitive performance was assessed by mapping several cognitive batteries available in the PRONIA study onto the six domains from the MATRICS consensus cognitive battery: social cognition, working memory, speed of processing, verbal learning, reasoning, and attention [50], [51], as described in the Supplementary Methods and Table S3.

We tested for neurocognitive, as well as sociodemographic and clinical group-level differences between HC individuals and ROP patients (regardless of subgroup) using, for continuous variables, two-sample t-tests or Mann Whitney U test in case the assumption of normality was not fulfilled, and, for binary variables, *X*^2^ test. We further tested for group differences between HC individuals and each subgroup, employing the same tests with Benjamini-Hochberg correction for the number of tests conducted for each variable (i.e., the number of subgroups). Lastly, we compared the ROP subgroups against each other in terms of the same variables, and the distribution of different ICD-10 diagnoses within each group, using ANOVA or Kruskal-Wallis and *X*^2^ tests. Post-hoc comparisons for ROP-subgroups were conducted using Tukey HSD or Dunn tests.

### 2.5 Subgroup characterization: Neuroimaging data analysis

Spatially smoothed GMV images were corrected for site/batch effects using a MATLAB-based implementation of the ComBat algorithm [52], [53] while preserving the effect of the patient subgroups. Then, corrected images were compared using a non-parametric permutation-based inference strategy as implemented in the Permutation Analysis of Linear Models software (PALM, a119, [54] running in MATLAB R2020b [55]). Statistical contrasts included bidirectional tests between HC individuals and all ROP patients, between HC individuals and each ROP factor subgroup individually, and between each pair of ROP subgroups. Additionally, we conducted a conjunction test computed as the maximum of the *P*-values of individual contrasts to identify signatures common for all ROP subgroups relative to the HC individuals. Mean-centered total-intracranial volume, age, and sex were entered as covariates into the design matrix. To ensure the tests’ validity, we defined exchangeability blocks for each site and performed within-site permutations for each contrast [56]. Family-wise error correction across the whole brain was performed using synchronized permutations [57], [58] of the threshold-free-cluster-enhancement [59] of the unpooled variance Welch t-statistic. We performed 1000 permutations and approximated the tail of the maximum permutation distribution using a generalized Pareto distribution [57]. The significance level was defined at *P*_FWE_<.05. Lastly, we quantified the percentage of statistically significant voxels within large-scale brain networks as defined based on the YEO 7 Networks atlas [60] and the Buckner atlas for cerebellum parcellation [61].

### 2.6 Subgroup characterization: Genotyping and Polygenic Risk Score calculation

Associations of PRS with overall ROP status and each ROP subgroup individually were estimated using binary logistic regression models (with HC individuals as reference) adjusted for sex and the first ten ancestry-informative principal components. Nagelkerke’s pseudo-R^2^ was derived and corrected for the covariates by substituting the null model in Nagelkerke’s equation for the model, including the covariates. The corrected pseudo-R^2^ obtained was then re-scaled to the liability scale [62], obtaining a value directly comparable with heritability and robust against ascertainment bias. A linear transformation on the liability scale was based on lifetime risk (*K* = .03 for overall ROP status [63], and for each subgroup, *K* = .03 was proportionally adjusted to the relative group size with a factor of .25. Within each set of analysis, Benjamini-Hochberg correction was employed to account for the assessed PRS *P*-thresholds.

### 2.7 Subgroup characterization: Longitudinal functioning trajectories

We employed linear mixed-effects models to account for the correlated nature of repeated measures within participants to investigate the association between the ROP subgroup and functioning across the follow-up time points. The models included fixed effects for subgroup membership, time point, age, and sex, with subject ID incorporated as a random effect. Functioning outcomes, including current Global Functioning Scale [64] ratings for Role (GF:Role) and Social (GF:Social), were considered as dependent variables. Primary outcomes focused on the coefficients associated with subgroups, indicating their impact on the specified dependent variables. All models were estimated using the lmer function from the lme4 package in R [65] after assessing the assumptions of the mixed-effects models, including checks for collinearity, residuals normality, and homoscedasticity. Additionally, pairwise contrasts between the ROP subgroups were analyzed using the emmeans package in R [66] with Tukey p-value adjustment for multiple comparisons. To further examine differential associations of subgroup membership and functioning over time, we added an interaction effect of ROP subgroup and timepoint to the models.

## 3. Results

### 3.1 Optimal factor solution

At T0, a four-factor model performed the best, yielding the minimum mean BIC (*BIC*[95%-CI]=−1195[−1195,−1194]) as determined by jackknife resampling (see Figure S2). The model’s factors yielded cumulative variances of *s* = 13, 22, 30, and 36%. The items with the highest loading on each factor were “lack of spontaneity”, “blunted affect”, and “poor rapport” on the first factor (in the following referred to as *Motor/Cognition* factor), “passive/apathetic social withdrawal”, “active social avoidance”, and “emotional withdrawal” on the second factor (*Social Withdrawal* factor); “excitement”, “lack of judgment and insight”, and ”unusual thought content” on the third factor (*Positive* factor); and “anxiety”, “tension”, and “depression” on the fourth factor (*Affective* factor; see Table S4). The internal consistencies of the four symptom factors were a_standardized_=.68, .57, .75, and .53. For factor correlations and factor score correlations, see Table S5 and S6. We grouped patients based on their maximum factor loadings (see Figure S3) into four subgroups: ROP-MOTCOG (*Motor/Cognition*, N = 78), ROP-SOCWD (*Social Withdrawal*, N = 73), ROP-POS (*Positive*, N = 85), and ROP-AFF (*Affective*, N = 92). Among patients initially categorized as ROP-MOTCOG with full longitudinal data (N_T1_=48, N_T2_=21), 68.75% remained ROP-MOTCOG at T1 and 66.67% at T2. Among patients initially categorized as ROP-SOCWD with full data (N_T1_=40, N_T2_=26), 37.5% remained in the ROP-SOCWD group at T1 and 42.3% at T2. Among patients initially categorized as ROP-POS with full data (N_T1_=59, N_T2_=38), 8.47% remained ROP-POS at T1 and 5.26% at T2. Of the patients initially categorized as ROP-AFF with full data (N_T1_=62, N_T2_=38), 32.26% remained ROP-AFF at T1 and 28.57% at T2. For a full overview of the between subgroup shifts, see Table S7. Table S8 shows the correlations of factor scores between time points.

### 3.2 Sociodemographic and Clinical Group-Level Comparisons

All ROP subgroups displayed significantly fewer education years relative to HC individuals (ROP-MOTCOG: *W* = 25266, *P* < .001; ROP-SOCWD: *W* = 22537, *P* < .001; ROP-POS: *W* = 25777.5, *P* < .001; ROP-AFF: *W* = 27741.5, *P* < .001, Table 1), and higher percentages of psychiatric disorder history among first-degree relatives (ROP-MOTCOG: χ2 = 66.37, *P* < .001; ROP-SOCWD: χ2 = 77.52, *P* < .001; ROP-POS: χ2 = 67.33, *P* < .001; ROP-AFF: χ2 = 61.46, *P* < .001) and second-degree relatives (ROP-MOTCOG: χ2 = 7.04, *P* = .02; ROP-SOCWD: χ2 = 18.03, *P* < .001; ROP-POS: χ2 = 22.05, *P* < .001; ROP-AFF: χ2 = 7.22, *P* = .02)(see Table S9). Differences in sex distribution were observed, with higher rates of males in three subgroups (ROP-MOTCOG: χ2 = 10.99, *P* = .002; ROP-SOCWD: χ2 = 18.73, *P* < .001; ROP-POS: χ2 = 11.40, *P* < .001). All subgroups reported a significantly higher rate of head trauma without loss of consciousness relative to HC individuals (ROP-MOTCOG: χ2 = 15.13, *P* < .001; ROP-SOCWD: χ2 = 19.68, *P* < .001; ROP-POS: χ2 = 14.39, *P* < .001; ROP-AFF: χ2 = 18.70, *P* < .001). Furthermore, all ROP subgroups showed significantly lower scores in neurocognition than HC individuals (attention, reasoning, social cognition, processing speed, verbal learning, working memory, and global cognition; all *P* < .001, Table 1).

When comparing ROP subgroups against each other, a diverging sex distribution was observed ( 2(3) = 8.80, *P* = .032), with ROP-AFF containing proportionally more female patients than ROP-SOCWD (all post-hoc comparisons are presented in Table 1). Furthermore, ROP subgroups differed in illness duration (*D*(3) = 13.94, P = .003), with ROP-SOCWD showing significantly longer illness duration than ROP-POS and ROP-AFF. Medication intake differed between subgroups for CPZE-, OLA-, and SSRI-equivalents (*D*(3) = 12.73, P = .005; *D*(3) = 11.81, P = .008; *D*(3) = 17.94, *P* < .001) with ROP-AFF showing the highest intake across all medication categories. Neurocognitive group differences were found for reasoning (*D*(3) = 9.36, *P* = .025), processing speed (*D*(3) = 13.16, *P* = .004), and global cognition (*D*(3) = 10.47, *P* = .015), with ROP-MOTCOG showing the lowest cognitive performances (Table 1). Lastly, we found significant differences in the distribution of depressive episodes with psychotic symptoms (F32.3/F33.3) but no other ICD-10 diagnoses between the ROP subgroups, such that ROP-SOCWD and ROP-AFF had the highest proportion of F32.3/F33.3 diagnoses (Table S10).

### 3.3 Gray matter volume signatures of ROP subgroups

**Figure 1:**
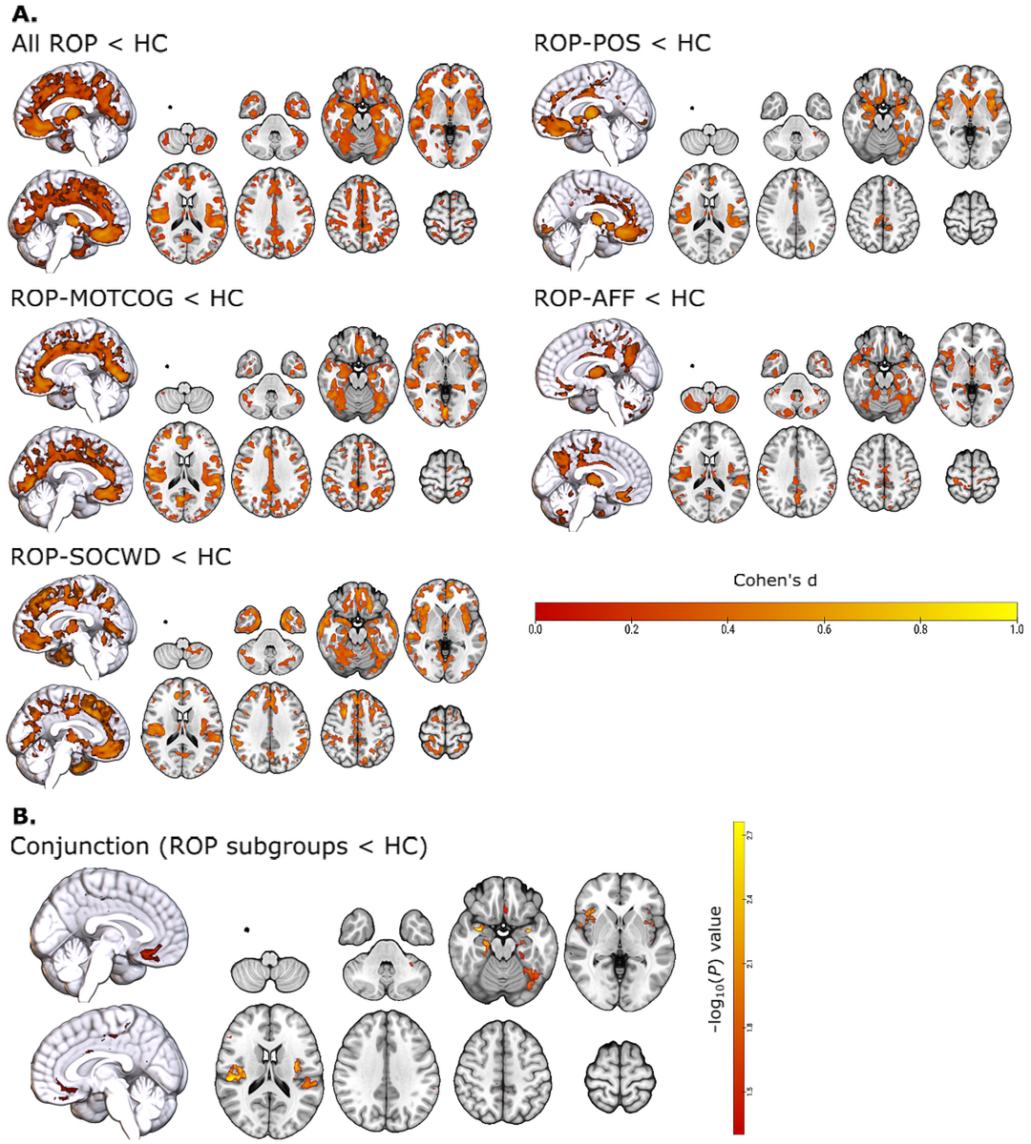
Significant GMV reductions in ROP patients relative to HC individuals at baseline. **(A)** Distribution of GMV patterns differentiating all ROP patients and each ROP patient subgroup from the HC individuals. Voxel-wise Cohen’s d values are shown for all voxels *P*_FWE_<.05 (family-wise error corrected for whole-brain) using the TFCE statistic. **(B)** Distribution of GMV differences that were common in differentiating each ROP patient subgroup from HC individuals, as determined using a conjunction test. The conjunction was calculated as the minimum −log_10_*P*_FWE_ value across the [ROP subgroup < HC individuals] contrasts and subsequently thresholded at *P*<.05, such that voxels that showed significant effects (family-wise error corrected for whole-brain) within all contrast were considered to be common GMV reductions across the ROP subgroups relative to HC individuals.

**Figure 2:**
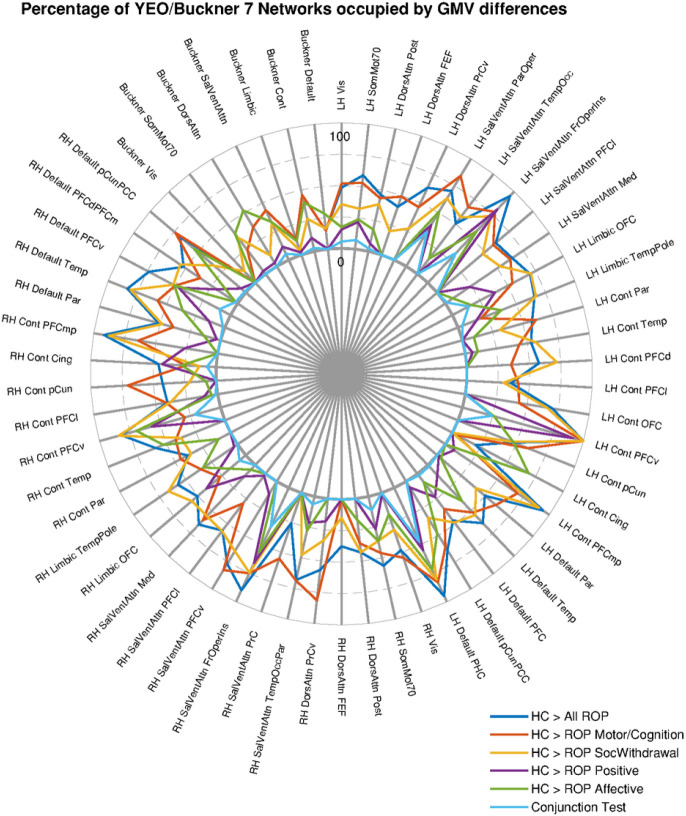
Regional distribution of the GMV patterns differentiating HC individuals from ROP patients across the entire sample and across PANSS-based subgroups. The percentage of statistically significant voxels was quantified within large-scale brain networks as defined based on the YEO 7 Networks atlas [60] and the Buckner atlas for cerebellum parcellation [61]. Only parcellations which had at least 1% of their volume occupied by the significant GMV patterns are displayed in the figure.

Independent of the PANSS-based symptom subgroup, all ROP patients showed significant GMV reductions across large-scale brain networks such as the control, default mode, and salience networks (Fig. 1A, 2) compared to HC individuals.

Regarding differences between each ROP subgroup and HC individuals, the ROP-MOTCOG subgroup was characterized by extended GMV reductions mainly within cingulate regions of the salience and control networks, as well as regions of the default mode, dorsal attention and limbic (orbitofrontal cortex) networks relative to HC individuals (Fig. 1A, 2). The ROP-SOCWD subgroup showed overlapping GMV decreases to the ROP-MOTCOG subgroup relative to HC; however, outside the cingulate gyrus and more extended in the medial prefrontal cortex (Fig. 1A, 2). The ROP-POS subgroup was characterized by GMV decreases mainly within the thalamus and anterior regions of the limbic (orbitofrontal cortex) and salience networks compared to HC individuals (Fig. 1A, 2). Lastly, the ROP-AFF subgroup showed GMV decreases in the thalamus, cerebellum, insula, and posterior regions of the control and default-mode network (Fig. 1A, 2). Furthermore, the conjunction analysis revealed GMV reductions within the salience (insula), default-mode (parahippocampus, PFC), limbic and frontotemporal regions within the control networks that were shared among all ROP subgroups relative to the HC individuals (Fig. 1B and 2). Finally, no significant GMV differences were found when comparing the ROP subgroups against each other.

### 3.4 Polygenic risk score characterization of ROP subgroups

**Figure 3:**
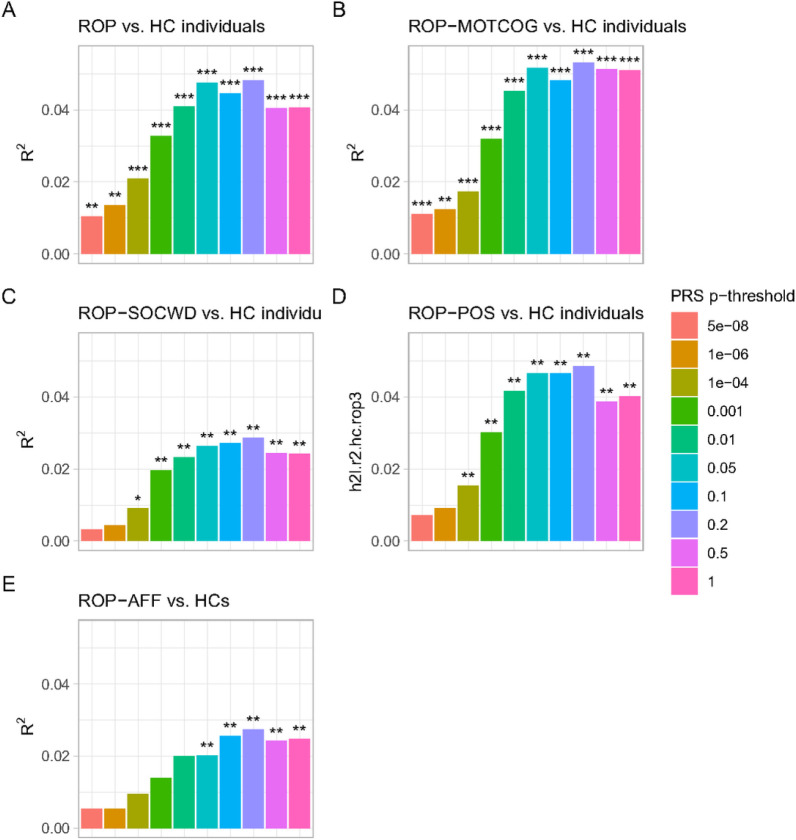
Phenotypic variance explained by PRS for schizophrenia for (A) all ROP patients, (B) ROP-MOTCOG, (C) ROP-SOCWD, (D) ROP-POS, (E) ROP-AFF. The stars shown above each bar intend to flag if the p-value of the association between ROP patients or ROP subgroup in the full model was below *P*<.001 (***), *P*<.01 (**), *P*<.05 (*). Bar colour represents PRS p-threshold.

The binary logistic regression analyses estimating the association between PRS for schizophrenia and a diagnosis of ROP, regardless of subgroup, revealed significant (*P* < .05) PRS effects across all 10 PRS thresholds (see Table S11). Irrespective of the threshold, a higher PRS increased the odds of ROP diagnosis. The PRS explained between 1.05–4.82% of the variance (re-scaled, corrected pseudo-R2) in ROP liability across thresholds (see Fig. 3). The PRS, including SNPs associated with SCZ at *P* < .2, explained 4.82% of the liability variance of ROP (*OR* = Inf, *P* < .001). The same PRS explained a similar amount of liability variance of the ROP-MOTCOG (5.32% variance explained, *OR* = Inf, *P* < .001) and ROP-POS subgroup (4.86% variance explained, *OR* = Inf, *P* < .001), and relatively smaller proportions of liability variance for ROP-SOCWI (2.86% variance explained, *OR* = Inf, *P* < .001), and ROP-AFF subgroup (2.74% variance explained, *OR* = Inf, *P* < .001) (see Fig. 3).

### 3.5 Longitudinal functioning trajectories of ROP subgroups

**Figure 4:**
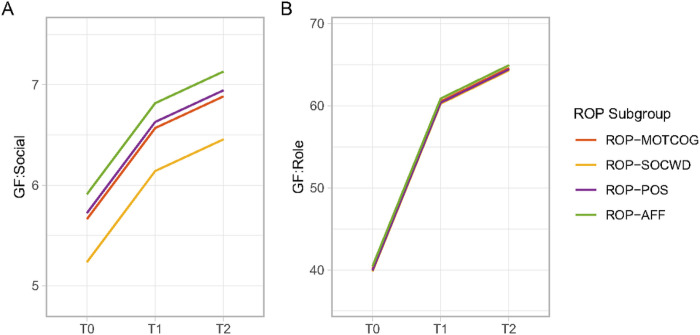
Longitudinal social and role function trajectory. The fixed effect of ROP subgroup on **(A)** GF:Social, and **(B)** GF:Role during follow-up in the linear mixed-effects models with no interaction terms between time point and ROP subgroup.

The mixed-effects model with GF:Social as the dependent variable without interaction between group and time point revealed a significant effect of the ROP subgroup (*F* = 3.53, *P* = .015, see Table S12), highlighting overall differences among the four groups. Similarly, the effect of time point was significant (*F* = 73.23, *P* < .001). When looking at the pairwise contrasts between the ROP subgroup throughout the whole follow-up period, ROP-SOCWD showed significantly lower scores in GF:Social than ROP-AFF (estimate=−0.68, *P* = .008, see Table S13). In the GF:Social model including the subgroup and time interaction, while significant ROP subgroup effects (*F* = 3.00, *P* = .030), time point effects (*F* = 70.52, *P* < .001) were found, the interaction between ROP group and time point was not statistically significant (*F* = .59, *P* = .740, see Table S12), suggesting that the impact of ROP subgroup on GF:Social did not change across different time points (see Fig. 4).

Neither of the GF:Role models revealed a significant effect of the ROP subgroup membership, indicating no overall differences among the four groups in GF:Role over the period (see Table S12). When including the interaction term between group and time, this effect was not significant, suggesting that the impact of the ROP subgroup on GF:Role did not change across time points (see Fig. 4). All model coefficients are depicted in Table S13.

## 4. Discussion

Early psychosis stages are characterized by diverse clinical manifestations, complicating the development of biologically informed diagnostic and treatment approaches [67]. In this context, a focus on clinical symptoms as proxies for potentially divergent pathophysiological pathways holds promise for advancing psychosis psychiatric care. Here, we provide novel evidence for distinct multimodal biological signatures underlying symptom-based subgroups within a multi-site longitudinal recent-onset psychosis (ROP) cohort.

Our data-driven factorization approach using symptom scores (PANSS) revealed four ROP subgroups with distinct clinical profiles characterized by *Motor/Cognition, Social Withdrawal, Positive*, and *Affective* symptoms. Our results are partially aligned with previous PANSS factorization studies, which identified between three and seven distinct factors in the early stages of psychosis [7], [9]–[13], [68], [69]. The variability across studies could result from different methodological approaches, sample composition, and statistical power. In this context, the optimal granularity of symptom dimensions for clinical translation may require evaluation relative to their mapping onto distinct biological pathways, and their relationship to prognostic and theragnostic outcomes.

Furthermore, we show moderate stability of the predominant symptom dimensions for the ROP patients over 18 months but also significant positive associations between the dimensions, which become stronger at the latest follow-up time point. These results support the idea of a crystallization of phenotypes along the disease trajectory [58], [59], [61]–[66] and a potential aggregation of the symptomatologic load over time, driven by the complex interactions and reciprocal loops between the brain systems underlying distinct symptoms.

Importantly, we found distinct biological signatures characterizing the ROP subgroups. The ROP-MOTCOG subgroup showed the lowest task-based processing speed and working memory performances, and extensive GMV decreases throughout the salience, default-mode, fronto-parietal networks, especially pronounced within the cingulate cortex. This pattern aligns with previous research showing gray matter volume reductions within the salience and control networks being associated with cognitive impairments in first-episode psychosis [28], [70]–[72]. The ROP-MOTCOG patients also showed the highest genetic liability for schizophrenia and the highest longitudinal stability of their subgroup membership from baseline to the follow-ups, suggesting a more “trait”-like symptom dimension in comparison to the other groups. Collectively, findings for the ROP-MOTCOG subtype align with the concept of “deficit schizophrenia”, characterized by severe and persistent cognitive deficits and structural alterations within frontotemporal brain systems [73]–[75].

The ROP-SOCWD subgroup showed overlapping GMV reductions with the ROP-MOTCOG subgroup outside the cingulate cortex and more extended to the medial prefrontal cortex, consistent with previous associations between medial prefrontal regions and emotion regulation and social cognition [76]–[78]. Additionally, this group showed the highest impairment of social functioning across time points, consistent with the socio-emotional nature of the negative symptoms included in this dimension. These results highlight the potential prognostic value of the ROP-SOCWD for poor social outcomes and encourage its further investigation within personalized stratification and intervention frameworks.

For the ROP-POS patients, we found GMV decreases in the thalamus, as well as limbic (orbitofrontal cortex) and salience networks, in line with previous reports of associations between positive psychotic symptoms and GMV reductions in the thalamus [24], [33], [34], and both functional and structural abnormalities in regions of the limbic [79], [80] and salience networks [81], [82]. Lastly, the ROP-AFF subgroup showed GMV decreases in the thalamus, cerebellum and posterior regions of the default-mode, salience and control networks, which have been previously linked to mood disorders [83]–[87].

Additionally, our results revealed GMV decreases in the fronto-temporal regions of the salience and control networks common among all ROP patient subgroups relative to the HC individuals, highlighting a potential “core” psychosis biomarker. This is consistent with previous reports of salience network abnormalities as a key finding across psychosis-spectrum disorders [81], [82], as well as studies highlighting structural fronto-temporal alterations independent of symptom-based subtypes in chronic schizophrenia patients [24].

While our study provides valuable insights, it’s important to acknowledge certain limitations. First, despite the suitability of subtyping approaches for clinical translation, the categorical assignment of subjects to factors based on their highest loading scores may impose hard boundaries to the continuous nature of psychiatric symptoms as proposed by modern nosological frameworks such as the RDoC [4], [88]. Future research may benefit from exploring the dimensional nature of the factors identified here, such that the exact clinical manifestations of the individual emanate from the interactions and aggregation of these distinct dimensions at each time point [89], [90]. Second, we have not explored subgroup-dependent longitudinal brain changes in the current analyses. Investigating whether early psychosis clinical subtypes are linked to divergent trajectories of brain volumetric degeneration/recovery would thus provide further insight into their prognostic value, as well as underlying pathophysiological mechanisms. Along similar lines, further research is required to establish the clinical validity and utility of such bio-behavioral subtypes by elucidating their individual-level prognostic value relative to clinically relevant outcomes such as daily functioning, treatment response or remission rates.

In conclusion, our findings support the existence of behavioral ROP subgroups characterized by distinct brain structural, genetic, neurocognitive and longitudinal functioning profiles. Our results highlight the potential of symptom-based subtyping for facilitating the development of biologically informed diagnostic, prognostic, and therapeutic strategies in early-stage psychosis.

## Figures and Tables

**Table 1:** Statistical comparison of sociodemographic, cognitive, and clinical characteristics between the HC individuals and ROP patients, as well as between the four ROP subgroups at baseline.

Variable	Whole groups				4-Factor Solution					
	HC	ROP	U/χ^2^ (*df*)	*P*	ROP-MOTCOG	ROP-SOCWD	ROP-POS	ROP-AFF	D/χ^2^ (*df*)	*P*
Sociodemographic										
N	464	328			78	73	85	92	–	–
Age (SD)	25.40 (6.01)	25.64 (5.89)	18071 5.50^MWU^	.600	25.37 (6.21)	26.00 (5.70)	25.31 (6.03)	26.05 (5.51)	2.40 (3)^D^	.494
Sex, male (%)	192 (41.38)	188 (58.57)	22.44 (4) ^2^	<.001	48 (61.54)	50 (68.49)	52 (61.18)	43 (46.74)	8.80 (3) ^2^	**.032** ^a^
Education, yrs (SD)	15.75 (3.25)	14.09 (7.02)	204671.00 ^2^	<.001	13.27 (3.24)	13.94 (2.96)	13.68 (3.42)	15.19 (12.01)	3.38 (3)^D^	.336
Clinical										
Family psychosis risk										
1st degree (%)	0 (0.00)	47 (14.69)	72.04 (4) ^2^	<.001	11 (14.10)	12 (16.44)	12 (14.29)	12 (13.04)	0.39 (3) ^2^	.942
2nd degree (%)	13 (2.83)	37 (11.64)	24.26 (4) ^2^	<.001	7 (8.97)	10 (13.70)	12 (14.63)	8 (8.70)	2.35 (3) ^2^	.502
Head Trauma (%)	36 (7.81)	73 (22.81)	35.41 (4) ^2^	<.001	17 (22.08)	18 (24.66)	18 (21.18)	21 (22.83)	0.29 (3) ^2^	.962
Birth Complications (%)	52 (11.28)	54 (16.93)	5.12 (4) ^2^	.024	11 (14.29)	16 (21.92)	15 (17.65)	14 (1 5.38)	1.82 (3) ^2^	.610
Onset Age (SD)		25.40 (5.89)			25.00 (6.34)	25.52 (5.62)	25.28 (5.94)	25.85 (5.60)	1.73 (3)^D^	.631
Illness Duration, years (SD)		0.50 (0.52)			0.51 (0.48)	0.70 (0.66)	0.37 (0.38)	0.43 (0.49)	13.94 (3)^D^	**.003** ^b^
Treatment										
Ever Psychotherapy (%)		269 (84.86)			70 (89.74)	59 (80.82)	68 (83.95)	79 (85.87)	2.52 (3) ^2^	.472
Ever Hospitalization (%)		228 (72.38)			61 (78.21)	47 (65.28)	56 (70.00)	68 (73.91)	3.43 (3) ^2^	.330
Ever Pharmacotherapy (%)		187 (59.55)			39 (50.00)	50 (69.44)	45 (56.96)	58 (63.04)	6.57 (3) ^2^	.087
Medication intake										
Antipsychotics* (CPZE-Equiv.)		682.73 (3524.32)			768.51 (2126.95)	389.67 (1147.56)	625.67 (2936.56)	947.77 (5594.99)	12.73 (3)^D^	**.005** ^c^
OLA-Equiv.*		23.39 (117.55)			26.27 (71.02)	13.56 (38.40)	21.37 (98.16)	32.34 (186.47)	11.81 (3)^D^	**.008** ^d^
Antidepressants* (SSRI-Equiv.)		19.50 (152.62)			768.51 (2126.95)	389.67 (1147.56)	625.67 (2936.56)	947.77 (5594.99)	17.94 (3)^D^	<.001^e^
Neurocognition										
Attention	0.45 (1.54)	−0.58 (1.79)	199581.00^MWU^	<.001	−0.07 (1.60)	0.19 (1.58)	0.08 (1.67)	−0.16 (1.94)	1.57 (3)^D^	.667
Reasoning	0.29 (0.90)	−0.36 (1.01)	186090.00^MWU^	< .001	−0.25 (0.98)	0.07 (0.85)	−0.05 (1.17)	0.20 (0.92)	9.36 (3)^D^	**.025** ^f^
Social Cognition	0.23 (0.84)	−0.25 (1.09)	194192.50^MWU^	< .001	−0.08 (0.99)	0.05 (1.06)	−0.13 (1.02)	0.15 (0.93)	4.78 (3)^D^	.188
Processing Speed	0.35 (0.56)	−0.46 (0.80)	198265.00^MWU^	< .001	−0.26 (0.77)	0.09 (0.82)	−0.02 (0.84)	0.16 (0.72)	13.16 (3)^D^	**.004** ^g^
Verbal Learning	0.35 (0.75)	−0.44 (1.09)	205331 .50^MWU^	< .001	−0.21 (1.00)	0.20 (0.94)	−0.02 (1.00)	0.05 (1.03)	6.83 (3)^D^	.077
Working Memory	0.31 (0.95)	−0.42 (0.89)	205466.00^MWU^	< .001	−0.26 (1.02)	0.17 (1.00)	0.09 (1.05)	0.00 (0.91)	5.50 (3)^D^	.139
Global Cognition	1.94 (3.36)	−2.51 (4.37)	178439.00^MWU^	< .001	−1.25 (4.41)	1.01 (3.59)	−0.23 (4.80)	0.50 (4.04)	10.47 (3)^D^	**.015** ^h^

*Note.* CPZE, Chlorpromazine; GF, Global Functioning; GAF; Global Assessment of Functioning (D/I, Disability and Impairment; S, Symptoms); HC, Healthy Control Individuals; SD, Standard Deviation; SSRI, Selective serotonin reuptake inhibitor; Significance threshold defined at *P* < .05.

* Cumulative Dosage [mg].

a ROP-AFF > ROP-SOCWD, *P* = .050

b ROP-SOCWD > ROP-POS, *P* = .003; ROP-SOCWD > ROP-AFF, *P* = .007

c ROP-MOTCOG > ROP-SOCWD, *P* = .003; ROP-AFF > ROP-MOTCOG, *P* = .032

d ROP-MOTCOG > ROP-SOCWD, *P* = .006; ROP-AFF > ROP-MOTCOG, *P* = .036

e ROP-MOTCOG > ROP-SOCWD, *P* = .003; ROP 3 > ROP-SOCWD, *P* = .007; ROP-AFF > ROP-MOTCOG, *P* = .008

f ROP-AFF > ROP-MOTCOG, *P* = .014

g ROP-SOCWD > ROP-MOTCOG, *P* = .010; ROP-POS > ROP-MOTCOG, *P* = .035; ROP-AFF > ROP-MOTCOG, *P* = .006

h ROP-SOCWD > ROP-MOTCOG, *P* = .015; ROP-AFF > ROP-MOTCOG; *P* = .036

## The PRONIA consortium

The following authors participated in the screening, recruitment, rating, examination, and follow-up of the study participants used in the current analyses. The authors are listed in alphabetical order according to the institution of affiliation.

**Department of Psychiatry and Psychotherapy, Ludwig-Maximilian-University, Munich, Germany:** Shalaila Haas, Alkomiet Hasan, Claudius Hoff, Ifrah Khanyaree, Camilla Krämer, Aylin Melo, Susanna Muckenhuber-Sternbauer, Yanis Köhler, Oemer Faruk Oeztuerk, Nora Penzel, David Popovic, Adrian Rangnick, Sebastian von Saldern, Rachele Sanfelici, Moritz Spangemacher, Ana Tupac, Maria Fernanda Urquijo, Johanna Weiske, Antonia Wosgien.

**Department of Psychiatry and Psychotherapy, University of Cologne, Cologne, Germany:** Karsten Blume, Dennis Hedderich, Dominika Julkowski, Nathalie Kaiser, Thorsten Lichtenstein, Ruth Milz, Alexandra Nikolaides, Tanja Pilgram, Mauro Seves, Martina Wassen.

**Department of Psychiatry (Psychiatric University Hospital, UPK), University of Basel, Switzerland:** Christina Andreou, Laura Egloff, Fabienne Harrisberger, Ulrike Heitz, Claudia Lenz, Letizia Leanza, Amatya Mackintosh, Renata Smieskova, Erich Studerus, Anna Walter, Sonja Widmayer.

**Institute of Mental Health & School of Psychology, University of Birmingham, United Kingdom:** Chris Day, Sian Lowri Griffiths, Mariam Iqbal, Mirabel Pelton, Pavan Mallikarjun, Alexandra Stainton, Ashleigh Lin.

**Department of Psychiatry, University of Turku, Finland:** Alexander Denissoff, Anu Ellilä, Tiina From, Markus Heinimaa, Tuula Ilonen, Päivi Jalo, Heikki Laurikainen, Antti Luutonen, Akseli Mäkela, Janina Paju, Henri Pesonen, Reetta-Liina Säilä, Anna Toivonen, Otto Turtonen.

**Department of Psychiatry (Psychiatric University Hospital LVR/HHU Düsseldorf), University of Düsseldorf, Germany:** Sonja Botterweck, Norman Kluthausen, Gerald Antoch, Julian Caspers, Hans-Jörg Wittsack.

**Department of Basic Medical Science, Neuroscience and Sense Organs - University of Bari Aldo Moro:** Giuseppe Blasi, Giulio Pergola, Grazia Caforio, Leonardo Fazio, Tiziana Quarto, Barbara Gelao, Raffaella Romano, Ileana Andriola, Andrea Falsetti, Marina Barone, Roberta Passiatore, Marina Sangiuliano.

**Department of Psychiatry and Psychotherapy of the University of Münster, Germany:** Marian Surmann, Olga Bienek, Udo Dannlowski.

General Electric Global Research Inc., USA.

Ana Beatriz Solana, Manuela Abraham, Timo Schirmer.

Workgroup of Paolo Brambilla, University of Milan, Italy:

Department of Neuroscience and Mental Health, Fondazione IRCCS Ca’ Granda Ospedale Maggiore Policlinico, University of Milan, Milan, Italy: Carlo Altamura, Marika Belleri, Francesca Bottinelli, Adele Ferro, Marta Re

Programma2000, Niguarda Hospital, Milan: Emiliano Monzani, Maurizio Sberna

San Paolo Hospital, Milan: Armando D’Agostino, Lorenzo Del Fabro

Villa San Benedetto Menni, Albese con Cassano (CO): Giampaolo Perna, Maria Nobile, Alessandra Alciati

Workgroup of Paolo Brambilla at the University of Udine, Italy.

Department of Medical Area, University of Udine, Udine, Italy: Matteo Balestrieri, Carolina Bonivento, Giuseppe Cabras, Franco Fabbro. IRCCS Scientific Institute “E. Medea”, Polo FVG, Udine: Marco Garzitto, Sara Piccin.

## References

[1] Fernandes B. S., Williams L. M., Steiner J., Leboyer M., Carvalho A. F., and Berk M., “The new field of ‘precision psychiatry,’” BMC Med., vol. 15, no. 1, p. 80, Apr. 2017. 28403846 10.1186/s12916-017-0849-x PMC5390384

[2] Friston K. J., “Precision psychiatry,” Biological psychiatry: cognitive neuroscience and neuroimaging, vol. 2, no. 8. Elsevier BV, pp. 640–643, Nov-2017. 29560899 10.1016/j.bpsc.2017.08.007

[3] American Psychiatric Association. Diagnostic and statistical manual of mental disoders..

[4] Insel T. , “Research domain criteria (RDoC): toward a new classification framework for research on mental disorders,” Am. J. Psychiatry, vol. 167, no. 7, pp. 748–751, Jul. 2010. 20595427 10.1176/appi.ajp.2010.09091379

[5] Kotov R. , “The Hierarchical Taxonomy of Psychopathology (HiTOP): A dimensional alternative to traditional nosologies,” J. Abnorm. Psychol., vol. 126, no. 4, pp. 454–477, May 2017. 28333488 10.1037/abn0000258

[6] Bassett A. S., Collins E. J., Nuttall S. E., and Honer W. G., “Positive and negative symptoms in families with schizophrenia,” Schizophr. Res., vol. 11, no. 1, pp. 9–19, Dec. 1993. 8297809 10.1016/0920-9964(93)90033-f PMC3188302

[7] Kemp K. C., Bathery A. J., Barrantes-Vidal N., and Kwapil T. R., “Positive, negative, and disorganized schizotypy predict differential patterns of interview-rated schizophrenia-spectrum symptoms and impairment,” Assessment, vol. 28, no. 1, pp. 141–152, Jan. 2021. 31955588 10.1177/1073191119900008

[8] Kay S. R., Fiszbein A., and Opler L. A., “The positive and negative syndrome scale (PANSS) for schizophrenia,” Schizophr. Bull., vol. 13, no. 2, pp. 261–276, 1987. 3616518 10.1093/schbul/13.2.261

[9] Langeveld J. , “Is there an optimal factor structure of the Positive and Negative Syndrome Scale in patients with first-episode psychosis?,” Scand. J. Psychol., vol. 54, no. 2, pp. 160–165, Apr. 2013. 23252448 10.1111/sjop.12017

[10] Best M. W., Grossman M., Oyewumi L. K., and Bowie C. R., “Examination of the Positive and Negative Syndrome Scale factor structure and longitudinal relationships with functioning in early psychosis,” Early Interv. Psychiatry, vol. 10, no. 2, pp. 165–170, Apr. 2016. 25277757 10.1111/eip.12190

[11] Martinuzzi E. , “Stratification and prediction of remission in first-episode psychosis patients: the OPTiMiSE cohort study,” Transl. Psychiatry, vol. 9, no. 1, Jan. 2019. 10.1038/s41398-018-0366-5 PMC6336802 30655509

[12] Tonna M. , “Dimensional structure of first episode psychosis,” Early Interv. Psychiatry, vol. 13, no. 6, pp. 1431–1438, Dec. 2019. 30644165 10.1111/eip.12789

[13] Jimeno N. , “Main symptomatic treatment targets in suspected and early psychosis: New insights from network analysis,” Schizophr. Bull., vol. 46, no. 4, pp. 884–895, Jul. 2020. 32010940 10.1093/schbul/sbz140 PMC7345824

[14] Yang Z., Lim K., Lam M., Keefe R., and Lee J., “Factor structure of the positive and negative syndrome scale (PANSS) in people at ultra high risk (UHR) for psychosis,” Schizophr. Res., vol. 201, pp. 85–90, Nov. 2018. 29804925 10.1016/j.schres.2018.05.024

[15] Amoretti S. , “Identifying clinical clusters with distinct trajectories in first-episode psychosis through an unsupervised machine learning technique,” Eur. Neuropsychopharmacol., vol. 47, pp. 112–129, Jun. 2021. 33531261 10.1016/j.euroneuro.2021.01.095

[16] Griffiths S. L. , “Structure and stability of symptoms in first episode psychosis: a longitudinal network approach,” Transl. Psychiatry, vol. 11, no. 1, p. 567, Nov. 2021. 34743179 10.1038/s41398-021-01687-y PMC8572227

[17] Petersen S. E. and Sporns O., “Brain networks and cognitive architectures,” Neuron, vol. 88, no. 1, pp. 207–219, Oct. 2015. 26447582 10.1016/j.neuron.2015.09.027 PMC4598639

[18] Bressler S. L. and Menon V., “Large-scale brain networks in cognition: emerging methods and principles,” Trends Cogn. Sci., vol. 14, no. 6, pp. 277–290, Jun. 2010. 20493761 10.1016/j.tics.2010.04.004

[19] Park H.-J. and Friston K., “Structural and functional brain networks: From connections to cognition,” Science, vol. 342, no. 6158, pp. 1238411–1238411, Nov. 2013. 24179229 10.1126/science.1238411

[20] Lepage M. , “Neurocognitive functions in persistent negative symptoms following a first episode of psychosis,” Eur. Neuropsychopharmacol., vol. 47, pp. 86–97, Jun. 2021. 33663901 10.1016/j.euroneuro.2021.02.008

[21] Ventura J., Hellemann G. S., Thames A. D., Koellner V., and Nuechterlein K. H., “Symptoms as mediators of the relationship between neurocognition and functional outcome in schizophrenia: a meta-analysis,” Schizophr. Res., vol. 113, no. 2–3, pp. 189–199, Sep. 2009. 19628375 10.1016/j.schres.2009.03.035 PMC2825750

[22] Rund B. R. , “Neurocognitive dysfunction in first-episode psychosis: correlates with symptoms, premorbid adjustment, and duration of untreated psychosis,” Am. J. Psychiatry, vol. 161, no. 3, pp. 466–472, Mar. 2004. 14992972 10.1176/appi.ajp.161.3.466

[23] Kravariti E. , “Linear and non-linear associations of symptom dimensions and cognitive function in first-onset psychosis,” Schizophr. Res., vol. 140, no. 1–3, pp. 221–231, Sep. 2012. 22766128 10.1016/j.schres.2012.06.008

[24] Koutsouleris N. , “Structural correlates of psychopathological symptom dimensions in schizophrenia: a voxel-based morphometric study,” Neuroimage, vol. 39, no. 4, pp. 1600–1612, Feb. 2008. 18054834 10.1016/j.neuroimage.2007.10.029

[25] Reichenberg A., “The assessment of neuropsychological functioning in schizophrenia,” Dialogues Clin. Neurosci., vol. 12, no. 3, pp. 383–392, 2010. 20954432 10.31887/DCNS.2010.12.3/areichenberg PMC3181984

[26] Kaplan R. D. , “Three clinical syndromes of schizophrenia in untreated subjects: relation to brain glucose activity measured by positron emission tomography (PET),” Schizophr. Res., vol. 11, no. 1, pp. 47–54, Dec. 1993. 8297804 10.1016/0920-9964(93)90037-j

[27] Liddle P. F., Friston K. J., Frith C. D., and Frackowiak R. S., “Cerebral blood flow and mental processes in schizophrenia,” J. R. Soc. Med., vol. 85, no. 4, pp. 224–227, Apr. 1992. 1433066 10.1177/014107689208500415 PMC1294730

[28] Lei W. , “Gray matter volume alterations in first-episode drug-naïve patients with deficit and nondeficit schizophrenia,” Psychiatry Res., vol. 234, no. 2, pp. 219–226, Nov. 2015. 26409573 10.1016/j.pscychresns.2015.09.015 PMC4859347

[29] Benoit A., Bodnar M., Malla A. K., Joober R., and Lepage M., “The structural neural substrates of persistent negative symptoms in first-episode of non-affective psychosis: a voxel-based morphometry study,” Front. Psychiatry, vol. 3, p. 42, May 2012. 22586412 10.3389/fpsyt.2012.00042 PMC3346965

[30] Bergé D., Carmona S., Rovira M., Bulbena A., Salgado P., and Vilarroya O., “Gray matter volume deficits and correlation with insight and negative symptoms in first-psychotic-episode subjects,” Acta Psychiatr. Scand., vol. 123, no. 6, pp. 431–439, Jun. 2011. 21054282 10.1111/j.1600-0447.2010.01635.x

[31] Ren W. , “Anatomical and functional brain abnormalities in drug-naive first-episode schizophrenia,” Am. J. Psychiatry, vol. 170, no. 11, pp. 1308–1316, Nov. 2013. 23732942 10.1176/appi.ajp.2013.12091148

[32] Bodnar M., Hovington C. L., Buchy L., Malla A. K., Joober R., and Lepage M., “Cortical thinning in temporo-parietal junction (TPJ) in non-affective first-episode of psychosis patients with persistent negative symptoms,” PLoS One, vol. 9, no. 6, p. e101372, Jun. 2014. 24979583 10.1371/journal.pone.0101372 PMC4076331

[33] Huang P. , “Decreased bilateral thalamic gray matter volume in first-episode schizophrenia with prominent hallucinatory symptoms: A volumetric MRI study,” Sci. Rep., vol. 5, no. 1, pp. 1–10, Sep. 2015. 10.1038/srep14505 PMC4585923 26403064

[34] Huang X. , “Decreased left putamen and thalamus volume correlates with delusions in first-episode schizophrenia patients,” Front. Psychiatry, vol. 8, Nov. 2017. 10.3389/fpsyt.2017.00245 PMC5702009 29209237

[35] Legge S. E. , “Associations between schizophrenia polygenic liability, symptom dimensions, and cognitive ability in schizophrenia,” JAMA Psychiatry, vol. 78, no. 10, pp. 1143–1151, Oct. 2021. 34347035 10.1001/jamapsychiatry.2021.1961 PMC8340009

[36] Xavier R. M., Dungan J. R., Keefe R. S. E., and Vorderstrasse A., “Polygenic signal for symptom dimensions and cognitive performance in patients with chronic schizophrenia,” Schizophr. Res. Cogn., vol. 12, pp. 11–19, Jun. 2018. 29552508 10.1016/j.scog.2018.01.001 PMC5852279

[37] Sengupta S. M. , “Polygenic Risk Score associated with specific symptom dimensions in first-episode psychosis,” Schizophr. Res., vol. 184, pp. 116–121, Jun. 2017. 27916287 10.1016/j.schres.2016.11.039

[38] Quattrone D. , “The continuity of effect of schizophrenia polygenic risk score and patterns of cannabis use on transdiagnostic symptom dimensions at first-episode psychosis: findings from the EU-GEI study,” Transl. Psychiatry, vol. 11, no. 1, p. 423, Aug. 2021. 34376640 10.1038/s41398-021-01526-0 PMC8355107

[39] R Core Team, “R: A Language and Environment for Statistical Computing,” R Foundation for Statistical Computing, Vienna, Austria, 2020.

[40] RStudio Team, “RStudio: Integrated Development Environment for R,” RStudio, PBC, Boston, MA, 2020.

[41] Revelle William, “psych: Procedures for Psychological, Psychometric, and Personality Research.” Northwestern University, Evanston, Illinois, 2023.

[42] Osborne J. W., Costello A. B., and Kellow J. T., “Best practices in exploratory factor analysis,” in Best Practices in Quantitative Methods, 2455 Teller Road, Thousand Oaks California 91320 United States of America: SAGE Publications, Inc., 2008, pp. 86–99.

[43] Hendrickson A. E. and White P. O., “Promax: A quick method for rotation to oblique simple structure,” Br. J. Stat. Psychol., vol. 17, no. 1, pp. 65–70, May 1964.

[44] Efron B. and Tibshirani R. J., “The jackknife,” in An Introduction to the Bootstrap, Boston, MA: Springer US, 1993, pp. 141–152.

[45] Schwarz G., “Estimating the dimension of a model,” Ann. Stat., vol. 6, no. 2, Mar. 1978.

[46] Babyak M. A., “What you see may not be what you get: A brief, nontechnical introduction to overfitting in regression-type models,” Psychosom. Med., vol. 66, no. 3, pp. 411–421, May 2004. 15184705 10.1097/01.psy.0000127692.23278.a9

[47] Friedl H. and Stampfer E., “Jackknife Resampling,” Encyclopedia of Environmetrics. Wiley, 31-Oct-2001.

[48] Ashburner J., “A fast diffeomorphic image registration algorithm,” Neuroimage, vol. 38, no. 1, pp. 95–113, 2007. 17761438 10.1016/j.neuroimage.2007.07.007

[49] Gaser C. and Dahnke R., “CAT-a computational anatomy toolbox for the analysis of structural MRI data,” Hbm, vol. 2016, pp. 336–348, 2016. 10.1093/gigascience/giae049 PMC11299546 39102518

[50] Nuechterlein K. H. , “The MATRICS Consensus Cognitive Battery, part 1: test selection, reliability, and validity,” Am. J. Psychiatry, vol. 165, no. 2, pp. 203–213, Feb. 2008. 18172019 10.1176/appi.ajp.2007.07010042

[51] Haas S. S. , “A multivariate neuromonitoring approach to neuroplasticity-based computerized cognitive training in recent onset psychosis,” Neuropsychopharmacology, vol. 46, no. 4, pp. 828–835, Mar. 2021. 33027802 10.1038/s41386-020-00877-4 PMC8027389

[52] Fortin J.-P. , “Harmonization of cortical thickness measurements across scanners and sites,” Neuroimage, vol. 167, pp. 104–120, Feb. 2018. 29155184 10.1016/j.neuroimage.2017.11.024 PMC5845848

[53] Johnson W. E., Li C., and Rabinovic A., “Adjusting batch effects in microarray expression data using empirical Bayes methods,” Biostatistics, vol. 8, no. 1, pp. 118–127, Jan. 2007. 16632515 10.1093/biostatistics/kxj037

[54] Winkler A. M., Ridgway G. R., Webster M. A., Smith S. M., and Nichols T. E., “Permutation inference for the general linear model,” Neuroimage, vol. 92, pp. 381–397, May 2014. 24530839 10.1016/j.neuroimage.2014.01.060 PMC4010955

[55] Matlab, version 9.9.0 (R2020b). Natick, Massachusetts: The MathWorks Inc., 2020.

[56] Winkler A. M., Webster M. A., Vidaurre D., Nichols T. E., and Smith S. M., “Multi-level block permutation,” Neuroimage, vol. 123, pp. 253–268, Dec. 2015. 26074200 10.1016/j.neuroimage.2015.05.092 PMC4644991

[57] Winkler A. M., Webster M. A., Brooks J. C., Tracey I., Smith S. M., and Nichols T. E., “Non-parametric combination and related permutation tests for neuroimaging,” Hum. Brain Mapp., vol. 37, no. 4, pp. 1486–1511, Apr. 2016. 26848101 10.1002/hbm.23115 PMC4783210

[58] Alberton B. A. V., Nichols T. E., Gamba H. R., and Winkler A. M., “Multiple testing correction over contrasts for brain imaging,” Neuroimage, vol. 216, no. 116760, p. 116760, Aug. 2020. 32201328 10.1016/j.neuroimage.2020.116760 PMC8191638

[59] Smith S. M. and Nichols T. E., “Threshold-free cluster enhancement: addressing problems of smoothing, threshold dependence and localisation in cluster inference,” Neuroimage, vol. 44, no. 1, pp. 83–98, Jan. 2009. 18501637 10.1016/j.neuroimage.2008.03.061

[60] Yeo B. T. T. , “The organization of the human cerebral cortex estimated by intrinsic functional connectivity,” J. Neurophysiol., vol. 106, no. 3, pp. 1125–1165, Sep. 2011. 21653723 10.1152/jn.00338.2011 PMC3174820

[61] Buckner R. L., Krienen F. M., Castellanos A., Diaz J. C., and Yeo B. T. T., “The organization of the human cerebellum estimated by intrinsic functional connectivity,” J. Neurophysiol., vol. 106, no. 5, pp. 2322–2345, Nov. 2011. 21795627 10.1152/jn.00339.2011 PMC3214121

[62] Lee S. H., Goddard M. E., Wray N. R., and Visscher P. M., “A better coefficient of determination for genetic profile analysis,” Genet. Epidemiol., vol. 36, no. 3, pp. 214–224, Apr. 2012. 22714935 10.1002/gepi.21614

[63] Perälä J. , “Lifetime prevalence of psychotic and bipolar I disorders in a general population,” Arch. Gen. Psychiatry, vol. 64, no. 1, p. 19, Jan. 2007. 17199051 10.1001/archpsyc.64.1.19

[64] Cornblatt B. A. , “Preliminary findings for two new measures of social and role functioning in the prodromal phase of schizophrenia,” Schizophr. Bull., vol. 33, no. 3, pp. 688–702, May 2007. 17440198 10.1093/schbul/sbm029 PMC2526147

[65] Bates D., Mächler M., Bolker B., and Walker S., “Fitting linear mixed-effects models Usinglme4,” J. Stat. Softw., vol. 67, no. 1, 2015.

[66] Lenth R., emmeans: Estimated Marginal Means, aka Least-Squares Means_. 2023.

[67] Chekroud A. M. , “Illusory generalizability of clinical prediction models,” Science, vol. 383, no. 6679, pp. 164–167, Jan. 2024. 38207039 10.1126/science.adg8538

[68] Salazar de Pablo G. , “Affective symptom dimensions in early-onset psychosis over time: a principal component factor analysis of the Young Mania Rating Scale and the Hamilton Depression Rating Scale,” Eur. Child Adolesc. Psychiatry, vol. 31, no. 11, pp. 1715–1728, Nov. 2022. 34052909 10.1007/s00787-021-01815-5

[69] Emsley R., Rabinowitz J., Torreman M., and RIS-INT-35 Early Psychosis Global Working Group, “The factor structure for the Positive and Negative Syndrome Scale (PANSS) in recent-onset psychosis,” Schizophr. Res., vol. 61, no. 1, pp. 47–57, May 2003. 12648735 10.1016/s0920-9964(02)00302-x

[70] Oomen P. P., Gangadin S. S., Begemann M. J. H., Visser E., Mandl R. C. W., and Sommer I. E. C., “The neurobiological characterization of distinct cognitive subtypes in early-phase schizophrenia-spectrum disorders,” Schizophr. Res., vol. 241, pp. 228–237, Mar. 2022. 35176721 10.1016/j.schres.2022.02.006

[71] Yasuda Y. , “Brain morphological and functional features in cognitive subgroups of schizophrenia,” Psychiatry Clin. Neurosci., vol. 74, no. 3, pp. 191–203, Mar. 2020. 31793131 10.1111/pcn.12963 PMC7065166

[72] Gallardo-Ruiz R., Crespo-Facorro B., Setién-Suero E., and Tordesillas-Gutierrez D., “Long-term grey matter changes in first episode psychosis: A systematic review,” Psychiatry Investig., vol. 16, no. 5, pp. 336–345, May 2019. 10.30773/pi.2019.02.10.1 PMC6539265 31132837

[73] Ahmed A. O., Strauss G. P., Buchanan R. W., Kirkpatrick B., and Carpenter W. T., “Schizophrenia heterogeneity revisited: Clinical, cognitive, and psychosocial correlates of statistically-derived negative symptoms subgroups,” J. Psychiatr. Res., vol. 97, pp. 8–15, Feb. 2018. 29156414 10.1016/j.jpsychires.2017.11.004

[74] Carruthers S. P., Van Rheenen T. E., Karantonis J. A., and Rossell S. L., “Characterising demographic, clinical and functional features of cognitive subgroups in schizophrenia spectrum disorders: A systematic review,” Neuropsychol. Rev., vol. 32, no. 4, pp. 807–827, Dec. 2022. 34694542 10.1007/s11065-021-09525-0

[75] Cascella N. G., Fieldstone S. C., Rao V. A., Pearlson G. D., Sawa A., and Schretlen D. J., “Gray-matter abnormalities in deficit schizophrenia,” Schizophr. Res., vol. 120, no. 1–3, pp. 63–70, Jul. 2010. 20452187 10.1016/j.schres.2010.03.039

[76] Kohn N., Eickhoff S. B., Scheller M., Laird A. R., Fox P. T., and Habel U., “Neural network of cognitive emotion regulation–an ALE meta-analysis and MACM analysis,” Neuroimage, vol. 87, pp. 345–355, Feb. 2014. 24220041 10.1016/j.neuroimage.2013.11.001 PMC4801480

[77] Bicks L. K., Koike H., Akbarian S., and Morishita H., “Prefrontal Cortex and Social Cognition in Mouse and Man,” Front. Psychol., vol. 6, Nov. 2015. 10.3389/fpsyg.2015.01805 PMC4659895 26635701

[78] Bzdok D. , “Segregation of the human medial prefrontal cortex in social cognition,” Front. Hum. Neurosci., vol. 7, p. 232, May 2013. 23755001 10.3389/fnhum.2013.00232 PMC3665907

[79] Walther S. , “Limbic links to paranoia: increased resting-state functional connectivity between amygdala, hippocampus and orbitofrontal cortex in schizophrenia patients with paranoia,” Eur. Arch. Psychiatry Clin. Neurosci., vol. 272, no. 6, pp. 1021–1032, Sep. 2022. 34636951 10.1007/s00406-021-01337-w PMC9388427

[80] Yang Y. , “Reduced gray matter volume in orbitofrontal cortex across schizophrenia, major depressive disorder, and bipolar disorder: A comparative imaging study,” Front. Neurosci., vol. 16, p. 919272, Jun. 2022. 35757556 10.3389/fnins.2022.919272 PMC9226907

[81] Palaniyappan L., Mallikarjun P., Joseph V., White T. P., and Liddle P. F., “Reality distortion is related to the structure of the salience network in schizophrenia,” Psychol. Med., vol. 41, no. 8, pp. 1701–1708, Aug. 2011. 21144116 10.1017/S0033291710002205

[82] Supekar K., Cai W., Krishnadas R., Palaniyappan L., and Menon V., “Dysregulated brain dynamics in a triple-network saliency model of schizophrenia and its relation to psychosis,” Biol. Psychiatry, vol. 85, no. 1, pp. 60–69, Jan. 2019. 30177256 10.1016/j.biopsych.2018.07.020

[83] Peng J. , “Cerebral and cerebellar gray matter reduction in first-episode patients with major depressive disorder: a voxel-based morphometry study,” Eur. J. Radiol., vol. 80, no. 2, pp. 395–399, Nov. 2011. 20466498 10.1016/j.ejrad.2010.04.006

[84] Wise T. , “Common and distinct patterns of grey-matter volume alteration in major depression and bipolar disorder: evidence from voxel-based meta-analysis,” Mol. Psychiatry, vol. 22, no. 10, pp. 1455–1463, Oct. 2017. 27217146 10.1038/mp.2016.72 PMC5622121

[85] Price J. L. and Drevets W. C., “Neural circuits underlying the pathophysiology of mood disorders,” Trends Cogn. Sci., vol. 16, no. 1, pp. 61–71, Jan. 2012. 22197477 10.1016/j.tics.2011.12.011

[86] Bora E., Harrison B. J., Davey C. G., Yücel M., and Pantelis C., “Meta-analysis of volumetric abnormalities in cortico-striatal-pallidal-thalamic circuits in major depressive disorder,” Psychol. Med., vol. 42, no. 4, pp. 671–681, Apr. 2012. 21910935 10.1017/S0033291711001668

[87] Sheline Y. I. , “The default mode network and self-referential processes in depression,” Proc. Natl. Acad. Sci. U. S. A., vol. 106, no. 6, pp. 1942–1947, Feb. 2009. 19171889 10.1073/pnas.0812686106 PMC2631078

[88] Casey B. J., Oliveri M. E., and Insel T., “A neurodevelopmental perspective on the research domain criteria (RDoC) framework,” Biol. Psychiatry, vol. 76, no. 5, pp. 350–353, Sep. 2014. 25103538 10.1016/j.biopsych.2014.01.006

[89] Feczko E., Miranda-Dominguez O., Marr M., Graham A. M., Nigg J. T., and Fair D. A., “The heterogeneity problem: Approaches to identify psychiatric subtypes,” Trends Cogn. Sci., vol. 23, no. 7, pp. 584–601, Jul. 2019. 31153774 10.1016/j.tics.2019.03.009 PMC6821457

[90] Ahmed A. O., Strauss G. P., Buchanan R. W., Kirkpatrick B., and Carpenter W. T., “Are negative symptoms dimensional or categorical? Detection and validation of deficit schizophrenia with taxometric and latent variable mixture models,” Schizophr. Bull., vol. 41, no. 4, pp. 879–891, Jul. 2015. 25399026 10.1093/schbul/sbu163 PMC4466177

